# Use of Hyaluronic Acid Hybrid Complex to Treat Wrinkles in the Upper Third of the Face: A Case Report

**DOI:** 10.7759/cureus.73451

**Published:** 2024-11-11

**Authors:** Daniel C Dziabas, Matheus Kasai, Gisele Chicone

**Affiliations:** 1 Dermatology, Dziabas Academy &amp; Research Institute, São Paulo, BRA

**Keywords:** aging, facial wrinkles, forehead, hyaluronic acid, hybrid hyaluronic acid complex, myomodulation, skin elasticity

## Abstract

Physical changes, such as skin atrophy, loss of elasticity, and the appearance of fine lines and wrinkles, are common during aging. A hybrid hyaluronic acid (HA) complex containing both low and high molecular weight has shown promising results in promoting skin restructuring by remodeling the epidermis, dermis, and hypodermis. This study describes three cases of facial aging, each with unique challenges. The upper third of the face was treated with two sessions spaced 30 days apart of hybrid HA using the blanching technique. These cases highlight the pressing need for alternative treatments that regenerate new tissues and cells, leading to a new skin architecture, as the use of neuromodulators in isolation can lead to unwanted side effects. The recovery of extracellular matrix proteins in this region with hybrid HA has significantly improved the elasticity, firmness, texture, and hydration of the skin, providing a healthier appearance and reducing the quantity and depth of facial wrinkles.

## Introduction

Skin aging is influenced by both intrinsic factors, affecting all tissues, and extrinsic factors, such as environmental damage from air pollution and UV radiation. This can lead to a decrease in skin integrity and physiological function, resulting in dryness, dysfunction, and increased risk of skin diseases and malignancy [1]. Recently, several interventions have emerged that aim to mitigate the signs of aging through therapies and techniques that provide more natural rejuvenation.

Complex physical changes occur during aging, with skin atrophy, loss of elasticity due to sagging skin, the appearance of spots and wrinkles, and resorption and displacement in soft tissues and bones, characterizing advancing age. In the aging of the forehead and glabella, we can observe the appearance of fine lines and wrinkles secondary to skin thinning and repeated muscle movements and a reduction in volume due to bone resorption and loss or thinning of fat compartments [2], altering the youthful convexity of the forehead.

The skin undergoes structural and molecular changes, in which a dehydrated epidermis becomes thinner at its dermal-epidermal junction, the villi become flattened, and the papillary dermis shows impaired fibroblast activity with a loss of response to growth factors and a decline in the production of extracellular matrix (ECM) proteins, with increased expression of proteases [1]. The elastic fibers decrease in number and diameter, and the reticular dermis becomes disorganized and degraded with fragmented fibers. This results in a loss of elasticity, turgor, and the consequent appearance of fine wrinkles, lines, and creases that are difficult to resolve, making them a therapeutic challenge [3].

The antagonist and agonist muscle groups work harmoniously to promote a youthful appearance. The lifters are more potent than the depressors in youth, but as we get older, their capacity is neutralized by the forces of gravity and the depressors, altering the facial structure [3]. For years, the upper third of the face was treated almost exclusively with neuromodulators, but using them in isolation can accentuate the ptosis of the eyebrows by weakening the frontalis muscle. However, the role of fillers have expanded; as they are inserted close to the muscles, they induce a mechanical blockade, which is myomodulation, i.e., it can alter the muscle movement, favoring the balance of facial contractility [4].

The recovery of dermal density in this region is necessary to improve the skin's elasticity, firmness, texture, and hydration. Hyaluronic acid (HA) is the main component of the ECM, a glycosaminoglycan that binds water molecules and is involved in tissue hydration but is markedly reduced in aging skin [5,6].

Treatment with HA promotes hydration of the dermis and helps combat the effects of aging, providing a healthier and longer-lasting appearance [7]. Hybrid HA, a remodeling agent that combines long and short chains [8], is not only safe but also highly effective. This biopolymer exhibits unique properties that allow for the creation of hydrogels that can mimic the natural ECM, promoting cell adhesion and differentiation in a controlled manner, which is crucial for optimal tissue regeneration in facial bioremodeling applications [9].

When administered intradermally in microdoses, it is safe from embolism and easy to inject. A BDDE-free (1,4-butanediol diglycidyl ether) product associated with intradermal application in microdoses is intended to guarantee that the injected product will maintain a safe plane from a vascular point of view, with low risk of adverse effects [10], making the technique safe. Its effectiveness in promoting tissue restructuring, improving skin texture and hydration, and myomodulation [11] instills confidence in its application, particularly in challenging areas such as the upper third of the face.

## Case presentation

Case 1

**Figure 1:**
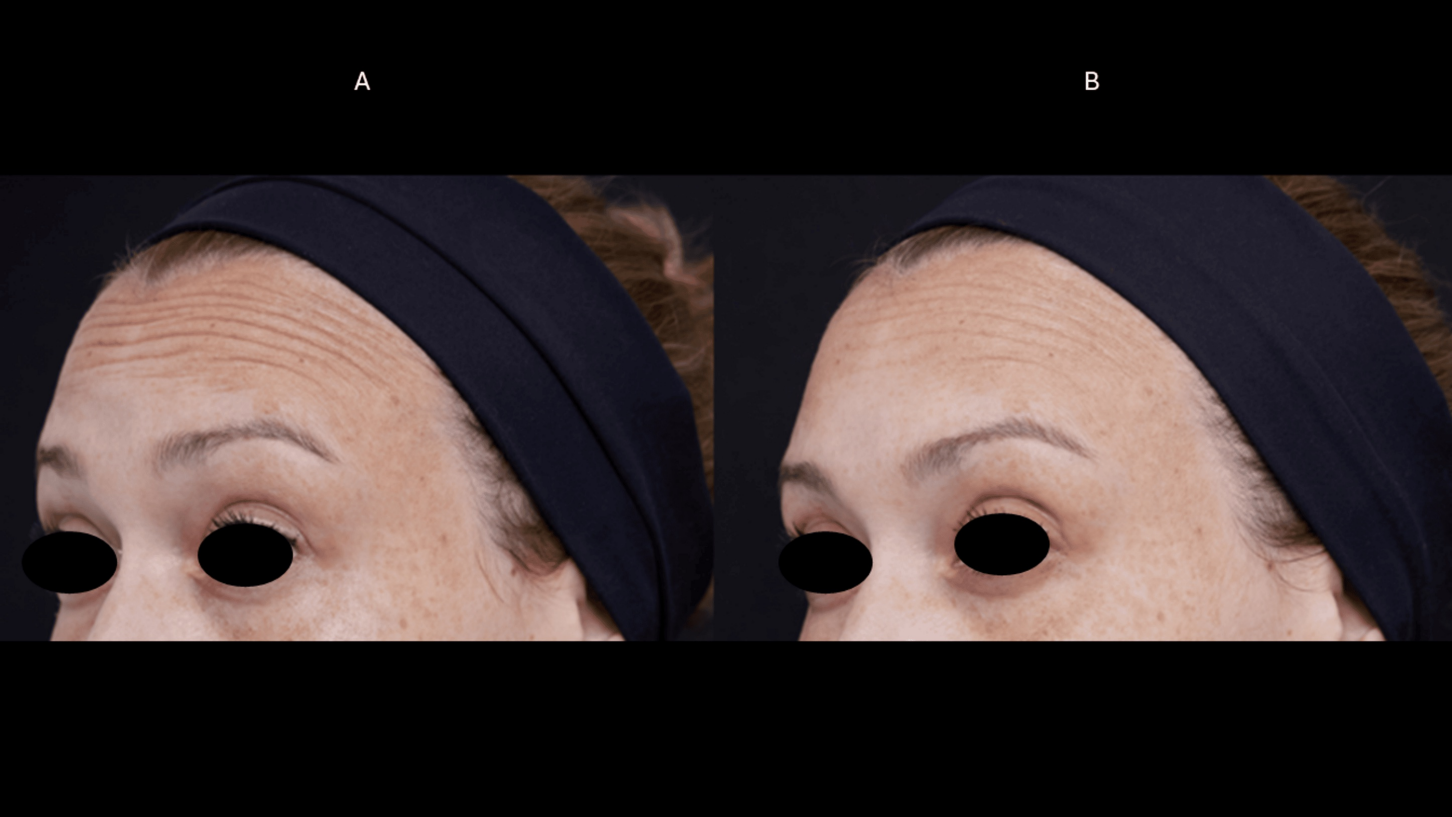
Frontal muscle myomodulation effect (case 1) Dynamic wrinkles in the forehead region before the treatment (A) and 60 days after treatment (B). Note the myomodulation of the frontalis muscle.

**Figure 2:**
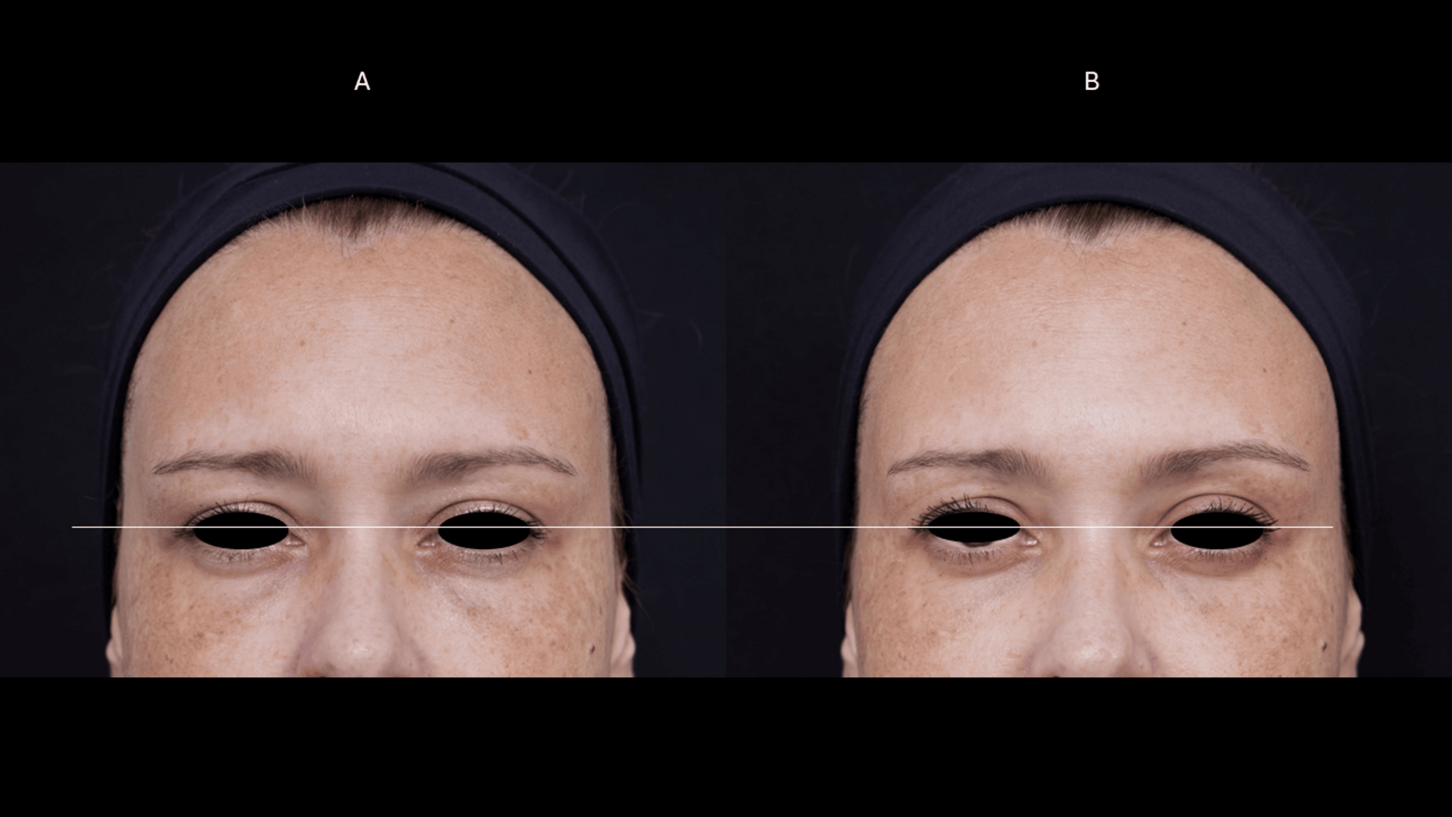
Eyelid ptosis (case 1) Mild eyelid ptosis before treatment (A) and presentation of eyelid retraction after the treatment (B).

A 35-year-old female, phototype 3, presented with bilateral static and dynamic wrinkles in the forehead and glabella, dynamic periocular wrinkles (crow's feet), and mild ptosis of the upper eyelids. Melasma affected the middle third and malar region. She had not undergone any previous aesthetic dermatological treatment in the last year, nor did she have botulin toxin, HA fillers, or collagen stimulators on her face. The patient had no significant past medical or surgical history and no family history of any dermatological disease (Figures 1, 2).

Case 2

**Figure 3:**
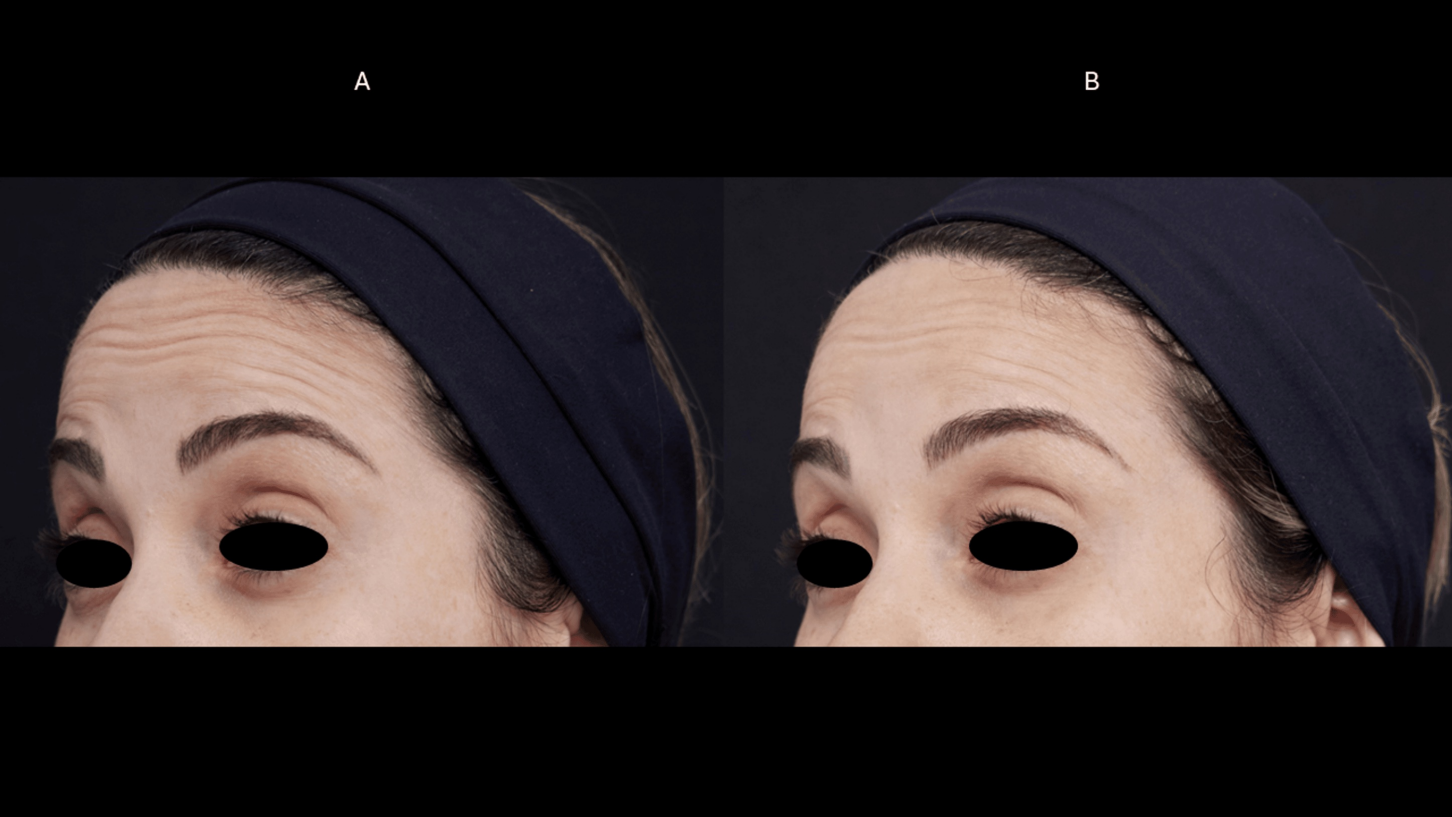
Frontal muscle myomodulation effect (case 2) Dynamic wrinkles in the forehead region before the treatment (A) and 60 days after treatment (B), with the myomodulation of the frontalis muscle and wrinkles reduction.

**Figure 4:**
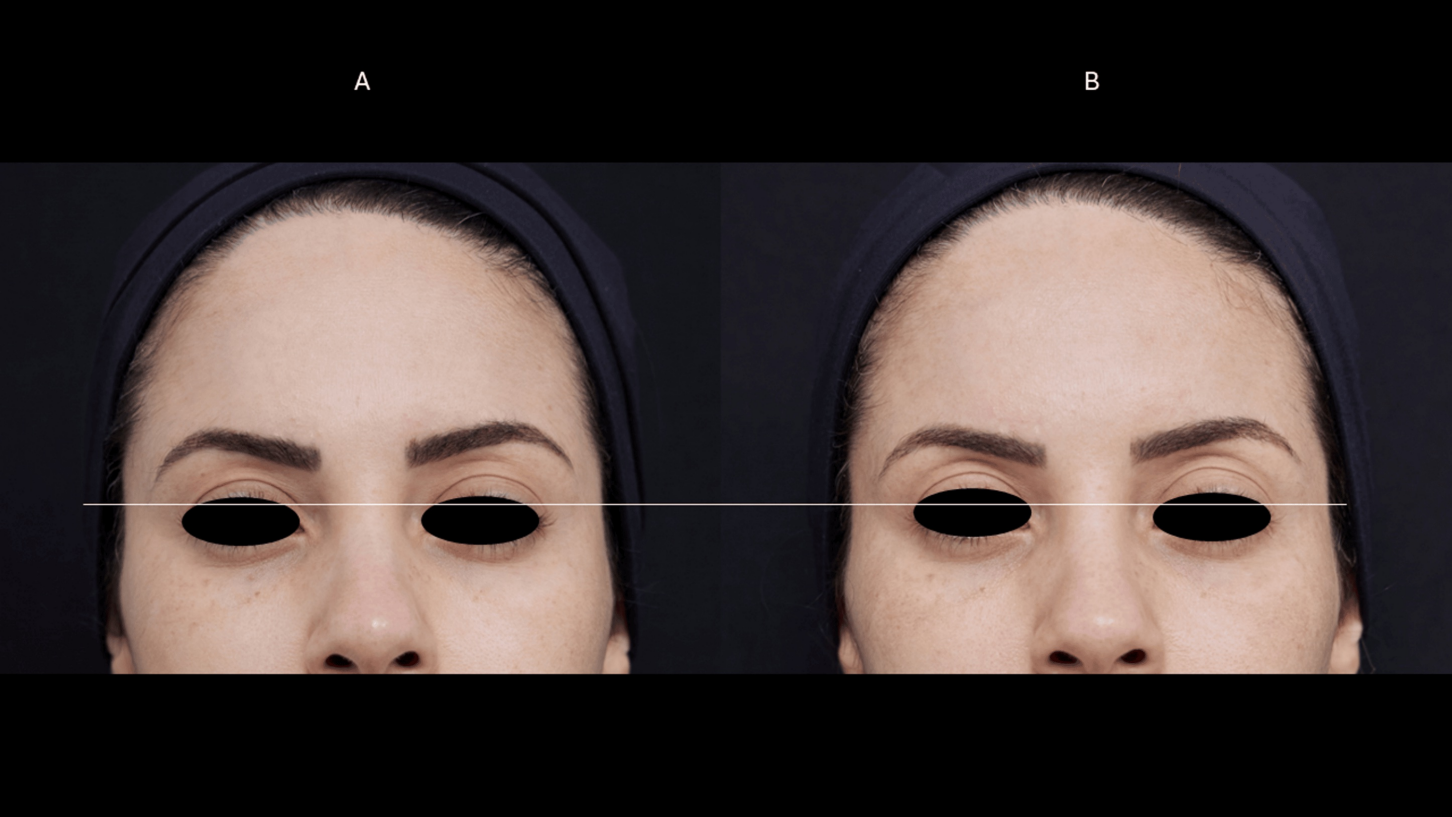
Eyelid ptosis (case 2) Mild eyelid ptosis before treatment (A) and presentation of eyelid retraction after the treatment (B).

A 37-year-old female, phototype 2, presented with dynamic wrinkles in the forehead, glabella, and periocular (crow's feet), as well as mild ptosis of the upper eyelids. The patient had never had botulinum toxin, HA filler, or any collagen-stimulating products. She had an atopic dermatitis controlled (Figures 3, 4).

Case 3

**Figure 5:**
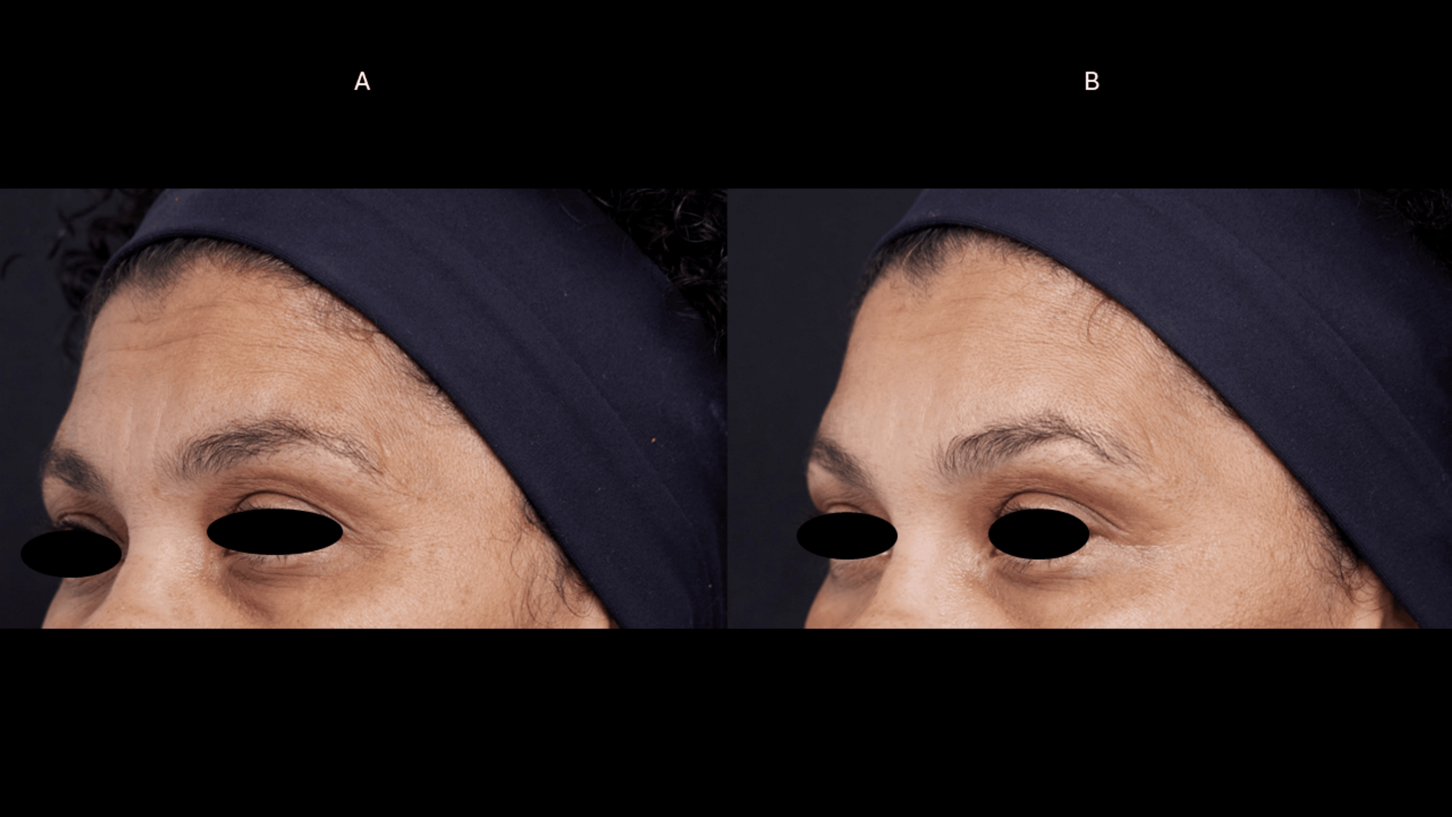
Frontal muscle myomodulation effect (case 3) Dynamic wrinkles in the forehead region before the treatment (A). There was a shallowing of dynamic wrinkles 60 days after treatment (B) with the myomodulation of the frontalis muscle and wrinkles reduction.

**Figure 6:**
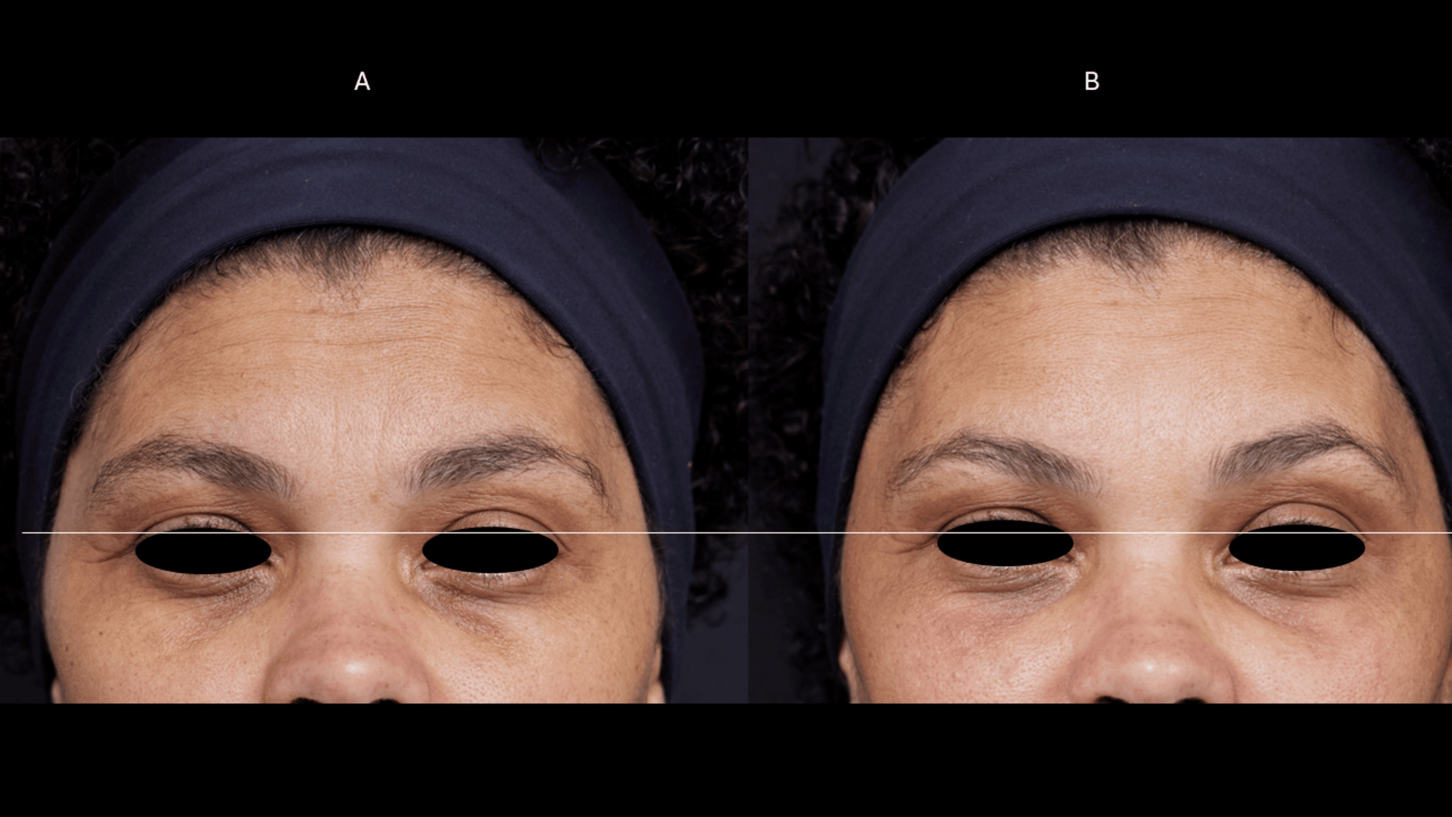
Eyelid ptosis (case 3) Moderate ptosis of the upper eyelids before treatment (A). There was eyelid retraction, reduction of static wrinkles, and lightening of hyperchromia 60 days after the treatment (B).

A 48-year-old female, phototype 4, presented with static wrinkles (dermal fractures) in the forehead, glabella, crow’s feet, and moderate ptosis of the upper eyelids, with the right more intense than the left. There were elastosis and hyperchromia in the upper and lower eyelids. She had not had any treatment with botulinum toxin, there was no residual action from HA fillers, and she had not used any collagen-stimulating products on her face. The patient had no significant past medical or surgical history and no family history of any dermatological disease (Figures 5, 6). 

This case report complies with the principles of the Declaration of Helsinki. Thus, authorization was given, and the patients signed the Free Informed Consent Form.

The proposed treatment was the intradermal administration of a hybrid HA complex in microdoses using the blanching technique. Two sessions were carried out 30 days apart, each using 2 mL of product to treat wrinkles, creases, and sagging in the upper third of the face. PROFHILO® Hybrid HA (IBSA - Institute Biochemical SA, Lugano, Switzerland) is a bio-remodeling agent containing 32 mg/mL of low-molecular-weight HA (80-100 Kda) and 32 mg/mL of high-molecular-weight HA (1,100-1,400 Kda). It features stable, hybrid, and cooperative complexes (HyCoCos) with low viscosity, produced using NAHYCO® Hybrid Technology, a thermal process that excludes using any chemical reagents [12]. The filling material is highly biocompatible and has a low viscosity, favoring optimum diffusion in the tissues to achieve remodeling of facial tissues [11].

**Figure 7:**
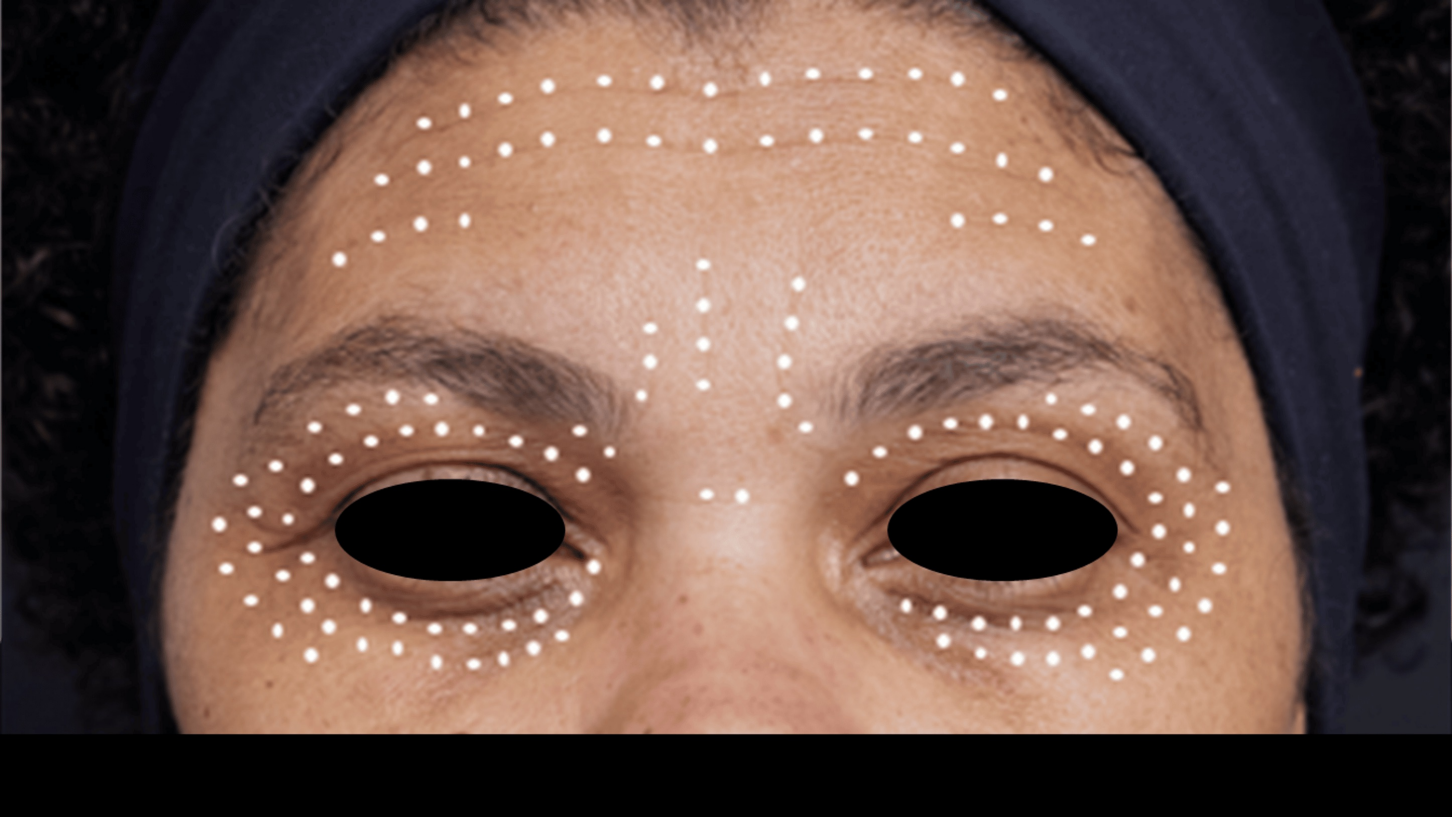
Hybrid hyaluronic acid microdoses applied with an intradermal blanching technique The microdoses were applied homogeneously: 1 mL in the forehead/glabella and 1mL in the eyelids (0.02 mL/point)

The microdoses were applied homogeneously to the upper third of the face, a crucial area affected by wrinkles, creases, and sagging, at points along the dynamic and static wrinkles, as well as on the upper and lower eyelids, with a space of 2 to 3mm between each point. Applications were carried out intradermally by inserting the angle of the 30G x 4mm needle at 10 to 15 degrees, i.e., practically parallel to the skin surface, allowing the needle to be seen. The product was injected until it formed a whitish micropapule. This technique was performed inside wrinkles on the forehead and glabella, as well as in the upper and lower eyelid area (Figure 7), playing a significant role in facial rejuvenation.

After the treatment, the patients were followed up, photos were taken, and a medical assessment was performed. All three treated patients showed an improvement in skin density, tone, and hydration. There were reduced static and dynamic wrinkles and eyelid retraction due to improved skin laxity. It should be noted that there were no documented reports of any treatment-related adverse events in all cases, except for slight edema, mainly in the lower eyelids, and a localized hematoma, which disappeared within two to seven days. Patient satisfaction results were particularly encouraging, with all patients reporting a score of 5 (exceptional improvement) on GAIS (Global Aesthetic Improvement Scale).

## Discussion

In assessing these cases, the safety of tissue rejuvenation treatment with high- and low-molecular-weight HA hybrid complexes, injected into the upper third of the face using the blanching technique, was a key finding in these cases, providing reassurance to both patients and practitioners.

Patients treated with high- and low-molecular-weight hybrid HA complexes showed a significant reduction in static and dynamic wrinkles, and a particularly promising improvement in tissue laxity due to eyelid retraction, offering a new avenue for rejuvenation.

HA, with its hygroscopic properties and biocompatibility, significantly improves skin hydration, a key factor in tissue restructuring, providing valuable insights for future treatments [13,14]. The gene expression of keratinocytes and fibroblasts is increased by using a hybrid complex of HA, leading to mRNA expression of type I and III collagen and elastin [15], as well as reducing transepidermal water loss and improving skin hydration. As a skin remodeler, it induces cell growth and differentiation by manipulating the components of the ECM, improving cell quality at all levels of the skin, and preventing or delaying senescence. HA binds to collagen fibers and elastin, giving the skin elasticity, tone, and resistance, thus improving wrinkles and sagging [16].

In addition, HA induces myomodulation, and when injected into areas close to the mimetic muscles, it causes a local mechanical blockade, acting as an antagonist. de Maio [4,17] has proposed that fillers be used as part of treatments to facilitate this myomodulation, applied directly below or above the muscle. Strategic and sequential placement of HA can influence muscle action and promote facial rejuvenation [18]. The complex of high- and low-molecular-weight HA has proven effective in clinical practice by reducing the signs of aging, acting as a restructuring, and stimulating the production of type I collagen through cell signaling by CD44 receptors [16,19]. Using the blanching technique [20], as described in other studies, and incorporating microdoses can reduce adverse effects, making it safer to use on the upper third of the face. Its action as a myomodulator has been observed clinically, as it provides mechanical blockade and reduces excessive muscle contraction, reducing dynamic wrinkles in patients. However, clinical intervention studies are urgent and crucial to prove and improve evidence-based clinical practice.

## Conclusions

In recent years, HA-based dermal bio remodelers have become the standard criterion in treating facial wrinkles and creases. The hybrid complex of high- and low-molecular-weight HA proved effective and safe in restoring vitality and turgor and reducing the signs of aging in the upper third of the face when applied with the blanching technique. The injectable treatment achieved good patient compliance and satisfaction with the result. These promising results could form the basis for further studies treating facial wrinkles.
